# Estimation of lifetime productivity loss from patients with chronic diseases: methods and empirical evidence of end-stage kidney disease from Taiwan

**DOI:** 10.1186/s13561-024-00480-z

**Published:** 2024-02-06

**Authors:** Fuhmei Wang, Jing-Shiang Hwang, Wen-Yen Huang, Yu-Tzu Chang, Jung-Der Wang

**Affiliations:** 1 Department of Economics in College of Social Science and Department of Public Health in College of Medicine, National Cheng Kung University, Tainan, Taiwan; 2 Institute of Statistical Science, Academia Sinica, Taipei, Taiwan; 3 Department of Public Health, College of Medicine, National Cheng Kung University, Tainan, 701 Taiwan; 4 Department of Internal Medicine, College of Medicine, National Cheng Kung University Hospital, National Cheng Kung University, Tainan, Taiwan; 5 Departments of Public Health and Occupational and Environmental Medicine, College of Medicine and Hospital, National Cheng Kung University, Tainan, Taiwan

**Keywords:** Societal burden, Productivity loss, End stage kidney disease, Presenteeism

## Abstract

**Objective:**

Studies that examine the broad allocation of resources, regardless of who bears the costs, should ideally estimate costs from a societal perspective. We have successfully integrated survival rates, employment ratios, and earnings to address the significant challenge of evaluating societal value through productivity assessments of patients with end-stage kidney disease (ESKD) in Taiwan.

**Methods:**

Using a theoretical framework, we interconnected two nationwide databases: the Taiwan National Health Insurance (NHI) and the Taiwan Mortality Registry from 2000 to 2017. Due to the statutory retirement age of 65, we collected data on all patients (83,358) aged 25–64 years diagnosed with ESKD and undergoing maintenance dialysis. We estimated the lifetime survival function through a rolling extrapolation algorithm, which was then combined with the monthly employment ratio and wages to calculate the lifetime employment duration and productivity up to the legal retirement age of ESKD patients. These were compared with sex-, age-, and calendar year-matched referents to determine the loss of employment duration and productivity of ESKD patients.

**Results:**

ESKD patients experienced a loss of approximately 25–56% in lifetime employment duration and a larger loss of about 32–66% in lifetime productivity after adjustments for different age, sex, and calendar year. The annual productivity loss per male (female) ESKD patient relative to that of the age-and calendar year-matched referent ranges from 75.5% to 82.1% (82.3% to 90.3%). During the periods when they are able to work (over the on-the-job duration) male ESKD patients lose between 34 and 56% of their income, and female ESKD patients lose between 39 and 68% of their income, compared to the age-and calendar year-matched referents. The loss of lifetime productivity is a combination of reduced lifetime employment duration, functional disability, absenteeism, and presenteeism at the workplace. The loss related to presenteeism is implied by the reduced wages.

**Conclusions:**

In addition to the loss of employment duration, we have empirically demonstrated the lifetime loss of productivity in patients with ESKD, also indicating the “presenteeism” resulted from inability to perform their job with full capacity over long-term periods.

**Supplementary Information:**

The online version contains supplementary material available at 10.1186/s13561-024-00480-z.

## Background

Research that explores the comprehensive allocation of resources should ideally calculate costs from a societal viewpoint, irrespective of who incurs these costs [1–3]. The total productivity loss over a lifetime can be seen as an economic production loss, potentially exceeding the medical costs of diseases. A detailed analysis of how poor health impacts the employment duration and productivity of working patients could provide a more accurate measure of the remaining human capital for survivors of related medical conditions. As an increasing number of patients with chronic diseases and complications are living longer, this issue has economic implications and warrants attention. Patients with end stage kidney disease (ESKD), undergoing maintenance dialysis, serve as an example. They can still contribute to the labor market, as explored in a previous study [4], although their reduced productivity while on the job has not been investigated. The costs of productivity loss resulting from morbidity and mortality are typically assessed through two methods in health economics: the human capital method (HCM) and the friction cost method (FCM), see [5–7], respectively. HCM estimates potential productivity loss due to disease over the expected remaining working life, but does not consider the risk of death. FCM estimates costs during the so-called friction period, which is the time it takes for a firm or organization to restore its original production level after losing a worker [8]. Both methods may not accurately reflect the true monetary values [9].

Most studies on the loss of earnings due to illness have been limited by their use of observational data and short time periods [6, 10]. To improve the accuracy of the lifetime survival extrapolation of an index cohort, external data from a reference population that matches in terms of age, sex, and calendar year can be used [11–13]. In this study, we develop a novel method to estimate the lifetime employment duration and real monthly wages of ESKD patients in Taiwan. These estimates can be combined with the survival function for our research objective on lifetime productivity estimation. As workers’ wages are a good representation of their productivities [14], we use wages for productivity estimation in this research.

This study builds upon previous research conducted by Chang et al. [4] using the same sample group of patients with ESKD. However, this study takes two major steps forward in quantifying the human capital loss resulting from chronic illnesses. Firstly, the model for extrapolation of employment ratio now includes mortality risks, which were not considered earlier. Secondly, this study has quantified the lifetime loss of earnings resulting from presenteeism. This study improves upon earlier studies in three ways: (1) It extrapolates estimates beyond the observable follow-up period of real data; (2) It evaluates the reduced productivity resulting from a combination of premature mortality, increased functional disability, and presenteeism at work; (3) It empirically reports the potential societal cost of ESKD under maintenance dialysis over a lifetime.

## Method

### Terms definitions

Lifetime productivity refers to the total economic output or value that an individual can produce over their working life. Employed-to-population ratio (EMRATIO) was defined as the ratio of individuals employed to the total concerned population. Presenteeism refers to the situation where an employee is physically present at work, but is not able to perform their job at full capacity over long-term periods due to their illness. Abstenteeism refers to instances where an employee is absent from work entirely due to their illness.

### The theoretical framework

#### Lifetime employment duration of an index cohort

In this study, we extend the method developed by Hwang et al. for estimating the lifetime medical costs of a specific study cohort [12]. Our extension allows us to estimate both lifetime employment duration and productivity, forming the basis of our theoretical model. We define ${e}_{i}^{c}\left(t\right)$ as a binary employment indicator for an individual $i$ in cohort $c$ at month $t$ after diagnosis, moreover ${a}_{i}$ denotes the onset age in years. We consider an individual’s employment from the time of diagnosis until they reach 65 years of age. Therefore, we set ${e}_{i}^{c}\left(t\right)=0$ for $t>\left(65-{a}_{i}\right)\times 12$. The age of 65 is the legal retirement age in Taiwan. Numerous factors can influence employment beyond this age. To simplify our model, we do not account for employment beyond the age of 65 for either cohorts (index and reference) when calculating productivity loss. We use *L* to represent the maximum possible duration in months that any individual within the index cohort could live post-diagnosis. An individual’s lifetime employment duration can then be expressed as ${\sum }_{t=0}^{L}{e}_{i}^{c}\left(t\right)$. The expected lifetime employment duration (*ELED*) of the cohort is calculated upon this:

1 $${ELED}_{c}=\frac{1}{N}{\sum }_{i=1}^{N}{\sum }_{t=0}^{L}{e}_{i}^{c}(t)={\sum }_{t=0}^{L}\left\{\frac{1}{N}{\sum }_{i=1}^{N}{e}_{i}^{c}(t)\right\}={\sum }_{t=0}^{L}\left\{\frac{{M}_{t}}{N}\times \frac{1}{{M}_{t}}{\sum }_{i\in {G}_{t}}{e}_{i}^{c}\left(t\right) + \frac{1}{N}{\sum }_{i\notin {G}_{t}}{e}_{i}^{c}(t)\right\}={\sum }_{t=0}^{L}S(t|c)\times E(t|c)$$

In which $N$ is the size of the cohort, ${G}_{t}$ is the subset of individuals still alive at month $t$, ${M}_{t}$ is the size of the subset, $S\left(t|c\right)={M}_{t}/N$ is the function of survival rate of cohort *c* at month *t*, $E\left(t|c\right)={\sum }_{i\in {G}_{t}}{e}_{i}^{c}\left(t\right)/{M}_{t}$ represents the employment rate relative to the cohort. $E\left(t|c\right)$ is called the conditional EMRATIO function. The product of the survival function $S\left(t|c\right)$ and the conditional EMRATIO function $E\left(t|c\right)$ of an index cohort *c* is referred to as the survival weighted EMRATIO function of the cohort. This measure is used to estimate the expected lifetime employment duration (*ELED*) of the cohort. The lifetime survival function $S\left(t|i\right)$ can be estimated using the method of survival extrapolation, which has been effectively used for estimating medical costs and quality of life in medical statistics [11, 12]. The conditional EMRATIO $E\left(t|i\right)$ of the index cohort beyond the follow-up can be extrapolated using the employment statuses of age-, sex-, and calendar year-matched referents from the general population.

#### Estimating the conditional EMRATIO function for the reference population

This study involved establishing the conditional EMRATIO function for the reference population through the use of real data from the National Health Insurance (NHI) registry in Taiwan. This was achieved by creating conditional employment tables of EMRATIO for each calendar year, y, years of age *a*, and sex *s*, which were denoted as $e\left(y,a,s\right)$. As mentioned earlier, we let $e\left(y,a,s\right)$ = 0 for $a>$ 65. To determine the life expectancy ${T}_{i}$ for a referent corresponding to the individual *i*. of age ${a}_{i}$ years, sex ${s}_{i}$, diagnosed year ${y}_{i}$ of the index cohort, life tables in Taiwan [15] were utilized. The expected employment status over time for the *i*^th^ referent was obtained as:

2 $$e_{it}\left(y,a,s\right)=e\left(y_i+\left\lfloor\frac t{12}\right\rfloor,a_i+\left\lfloor\frac t{12}\right\rfloor,s_i\right)\;\mathrm{for}\;0\leq t\leq T_i,$$

where the floor bracket $\lfloor u\rfloor$ denotes the largest integer that does not exceed *u*. The lifetime conditional EMRATIO function $\widehat{E}\left(t|r\right)$ given the reference cohort *r* was calculated by the average of the expected employment statuses of all the generated referents alive at time *t*, following [16]:

3 $$\widehat E\left(t\vert r\right)=\frac{\sum_ie_{it}\left(y,a,s\right)}{\sum_i\#\left\{i:{t\leq T}_i\right\}}\;\mathrm{for}\;0\leq t\leq L$$

#### Extrapolating the conditional EMRATIO function for the index cohort

The methodology used in this work to calculate the conditional EMRATIO function $E\left(t|i\right)$ for the index cohort involves direct computations up to the end of the follow-up period. To extrapolate $E\left(t|i\right)$ beyond the follow-up period, the lifetime survival function and the lifetime conditional EMRATIO function of the referents are utilized. When expressing the hazard function $h\left(t|c\right)$ of a cohort c using the Cox proportional model, factors such as employment status, age, and sex are taken into account. These factors are assumed to be associated with the hazard of death within the cohort as:

4 $$h\left(t|c\right)={h}_{0}\left(t\right)\times exp\left[{\mu }_{c}+{\alpha }_{1}g\left(E\left(t|c\right)\right)+{\alpha }_{2}\left(A\left(t|c\right)\right)+{\alpha }_{3}\left(M\left(t|c\right)\right)\right]$$

in which ${h}_{0}\left(t\right)$ is the baseline hazard function; $g\left(\cdot\right)$ is a transformation function; $E\left(t|c\right)$ denotes the conditional EMRATIO function of the cohort *c*; $A\left(t|c\right)$ represents the average age at time *t* in the cohort; $M\left(t|c\right)$ stands for the proportion of males at time *t* in the cohort. The hazard ratio between the index and reference groups equals $HR\left(t\right)=h\left(t|i\right)/h(t|r)$. After taking the log of $HR\left(t\right)$, we obtained:

5 $$log\left(HR\left(t\right)\right)={\mu }_{d}+{\alpha }_{1}{E}_{d}\left(t\right)+{\alpha }_{2}{A}_{d}\left(t\right)+{\alpha }_{3}{M}_{d}\left(t\right)$$

where ${E}_{d}\left(t\right)=g\left(E\left(t|i\right)\right)-g\left(E\left(t|r\right)\right)$ and the variables *A*_d_ and *M*_d_ were defined similarly. Using Eq. (5), we can rewrite the relationship between $HR\left(t\right)$ and ${E}_{d}\left(t\right)$ into a simple linear regression form:

6 $${E}_{d}\left(t\right)={\beta }_{0}+{\beta }_{1}log\left(HR\left(t\right)\right)+{\varepsilon }_{t}$$

In order to estimate the conditional EMATIO of the index group, we utilize the logit transformation $g\left(p\right)=\text{log (}p/\left(1-p\right)\text{)}$ on the interval (0, 1). The logit function is the inverse of the logistic function. Since the values of EMRATIO are between 0 and 1, the logit transformation allows these probabilities to be modeled on the whole real line. This involves using the lifetime estimate $\widehat{HR}\left(t\right)$ of hazard ratio, the lifetime estimate $\widehat{E}\left(t|r\right)$, and the estimate $\widehat{E}\left(t|i\right)$ for time *t* from some time *a* to the end of follow-up *F* to determine the regression model’s intercept and slope. Finally, we extrapolate the conditional EMATRIO of the index group:

7 $$\widehat E\left(t\vert i\right)=\frac{\text{exp}\{\text{logit}(\widehat E(t\vert r))+{\widehat\beta}_0+{\widehat\beta}_1\text{log}(\widehat{HR}(t))\}}{1+\text{exp}\{\text{logit}(\widehat E(t\vert r))+{\widehat\beta}_0+{\widehat\beta}_1\text{log}(\widehat{HR}(t))\}}\;\mathrm{for}\;t>F$$

The expected lifetime loss of employment duration (*LLED*) can then be estimated as:

8 $$\widehat{LLED}={\sum }_{0}^{L}[\widehat{S}(t|r)\times \widehat{E}(t|r)-\widehat{S}\left(t|i\right)\times \widehat{E}\left(t|i\right)]$$

#### Estimating the lifetime loss of earnings for an index cohort

We can derive a formula similar to Eq. (1) for calculating the expected lifetime earnings (*ELN*) of the index cohort:

9 $${ELN}_{c}={\sum }_{t=0}^{L}S\left(t|c\right)\times N\left(t|c\right)$$

where the function *N* $\left(t|c\right)$ represents the conditional average earnings of the individuals still alive in the index cohort at time t. By applying the same procedures outlines in Eqs. (2) through (6), and using the new transformation function $g\left(p\right)=log\left(p\right)$, we can extrapolate the conditional average earnings of the index group as follows:

10 $$\widehat N\left(t\vert i\right)=\exp\{\log(\widehat N\left(t\vert r\right))+{\widehat\beta}_0+{\widehat\beta}_1\log(\widehat{HR}(t))\}\;\mathrm{for}\;t>F$$

where the function $\widehat{N}\left(t|r\right)$ represents the conditional average earnings for the reference group. The expected lifetime loss of productivity (LLEP) for the index cohort can then be estimated as:

11 $$\widehat{LLEP}={\sum }_{0}^{L}[\widehat{S}(t|r)\times \widehat{N}(t|r)-\widehat{S}\left(t|i\right)\times \widehat{N}\left(t|i\right)]$$

Equation (11) serves as a predictive model for the anticipated loss of productivity over the lifetime of a patient suffering from a major catastrophic illness, even while they continue to work. This equation takes into account the dynamic fluctuations in wages, which can serve as a significant indicator of presenteeism. In the context of patients with major catastrophic illness, even though they may still be employed, their productivity or contribution to the labor market is negatively affected by their health condition. This impact is often reflected in the changes of their wages.

### Data and statistical analysis

This research was initiated following the receipt of grant support from the National Science and Technology Council (Grant numbers: NSTC 111–2627-M-006–003, 112–2627-M-006–002, 112–2410-H-006–105-). This study was also approved by the Institutional Review Board of National Cheng Kung University Hospital in Taiwan (Approval Number: IRBB-ER-105–386). It should be noted that no participant received compensation or was offered any incentive for their participation in this study.

In order to conduct a comprehensive analysis, we combined data from two large-scale databases in Taiwan: The National Health Insurance (NHI) and the Mortality Registry. The NHI database includes demographic information, reimbursement records for clinical diagnoses, and premium collections based on the monthly salary of various types of workers: civil servants, private sector employees, employers, unemployed individuals, and dependents. The Mortality Registry, on the other hand, contains data on the cause of death and date of death. To ensure privacy, the personal identification codes were encrypted. We also utilized the national life tables from Taiwan’s vital statistics to determine the survival time of the reference group over their lifetime.

We established our index cohort of patients with ESKD by identifying individuals from the NHI research database. These patients began receiving treatment with maintenance dialysis therapy [coded as ICD-9-CM) 585 or (ICD-CM-10) N18.6] at a time between 2000 and 2017. They were between 25 and 64 years of age. Given that our extrapolation method requires a large sample size for accuracy, we stratified age groups into 10-year intervals for those under 35, and 5-year intervals for those older. We simplified the analysis by assuming that all individuals over 65 were retired, thereby excluding them from the employment duration and productivity analysis outlined in Eqs. (1)–(11). The EMRATIOs of individuals aged ≥ 65 were assumed to be 0. We implemented the extrapolation algorithm to estimate the lifetime survival function and life expectancy of index cohort using the R package iSQol2, available at http://sites.stat.sinica.edu.tw/isqol/.

Our research involved data collection from the catastrophic registry for patients diagnosed with ESKD between 2000 and 2017 (186,335). We excluded patients with missing sex data (282), missing birth date (308), outside the age range of 25 to 64 (97,044), cases where the date of death preceded the diagnosis date (176), and patients with cancer (4,802) or who received a kidney transplant before dialysis (365). This left us with 83,358 eligible patients for the study. To ensure the reliability of our analysis, we excluded data points for any month where the employment ratio was based on fewer than 50 patients in the study group [4]. This helped us minimize the impact of outliers that could skew our results due to a small sample size at the end of the follow-up period. The insurance premiums for the employed were calculated according to monthly earnings, which excluded year-end bonuses. We found a cap of USD 6070 in 2017. The average exchange rate over the period from 2000 to 2017 was Taiwan New Dollars (TWD) 31.95 to 1 USD. Utilizing the longitudinal database of vital statistics and the hazard functions from life tables for calendar years in Taiwan, we constructed the lifetime survival functions for the reference group, which were matched according to age, sex, and calendar year. By applying similar methods, we found that the estimated life expectancy for the seven age strata of the male (female) reference groups were generally greater than those of the corresponding index cohorts.

## Results

Table 1 provides a demographic overview of the index cohort of ESKD patients diagnosed at the beginning of follow-up. In the labor market, individuals under 15 years of age are not permitted to work. In Taiwan, individuals graduate from colleges around the age of 22, and many continue to graduate school before entering the workforce, remaining employed until the legal retirement age of 65. In this study, over the period from 2000 to 2017, the EMRATIOs of the index cohort for males and females increased with age, peaking at approximately 0.82 and 0.75, respectively, for the 51–55 age group. After this peak, the EMRATIO decreased with age until 64, dropping to around 0.5 for males and 0.4 for females.

**Table 1:** Demographic characteristics of the index cohort with end stage kidney disease (ESKD) at the beginning of maintenance dialysis (83,358)

	**Male (47,622)**							**Female (35,736)**						
**Age**	**25–34**	**35–40**	**41–45**	**46–50**	**51–55**	**56–60**	**61–64**	**25–34**	**35–40**	**41–45**	**46–50**	**51–55**	**56–60**	**61–64**
**Total**	2,548	3,365	4,520	7,202	9,953	11,092	8,942	2,142	2,518	3,384	5,150	6,607	8,162	7,773
**Employment status, N (%)**														
Employed	1,501 (59)	2,117 (63)	2,889 (64)	4,750 (66)	6,176 (62)	5,973 (54)	3,968 (44)	1,181 (55)	1,554 (62)	2,174 (64)	3,380 (66)	4,127 (62)	3,831 (47)	2,962 (38)
Unemployed	1,047 (41)	1,248 (37)	1,631 (36)	2,452 (34)	3,777 (38)	5,119 (46)	4,974 (56)	961 (45)	964 (38)	1,210 (36)	1,770 (34)	2,480 (38)	4,331 (53)	4,811 (62)
**Monthly working salary in baseline (USD)**^**a**^														
ESKD patients	490	602	616	638	606	536	410	424	493	514	519	500	388	296
Reference	796	954	963	957	921	799	587	669	703	693	699	668	531	366

^a^Based on the mean exchange rate over the period from 2000 to 2017: 1 USD = 31.95 TWD

As shown in Table 1, the monthly earnings for reference males and females increased with age, peaking at USD 1333 and USD 1000 at ages 44 and 50, respectively. After this peak, the earnings decreased with age, reaching a low of USD 334 for males and USD 200 for females at age 64. For all age groups, the earnings of the reference individuals were consistently higher than those of the ESKD patients. The EMRATIO function and the average salary stratified by sex, age, and calendar year for the reference group are presented in Supplementary Figures 1 and 2.

### Estimation of lifetime employment duration

**Fig. 1:**
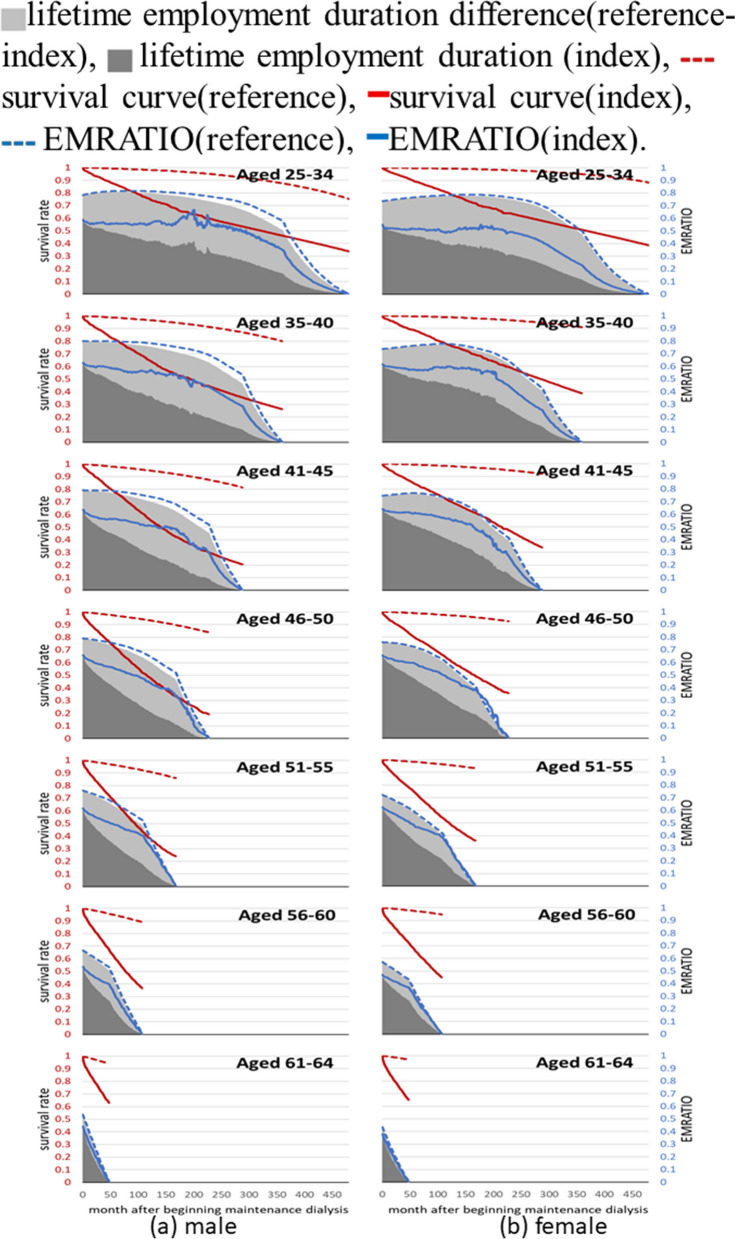
Expected lifetime employment duration of male ESKD patients and the corresponding age-sex-, and calendar year-matched referents, stratified by age

Equation (1) was used to calculate the lifetime employment duration for seven different age groups of male and female patients in the index group. This was achieved by multiplying the lifetime survival function by the extrapolation of EMRATIO. Table 2 displays the life expectancies, the lifetime employment duration, and the relative loss of LED for patients with end stage kidney disease (Index) and their corresponding age-, sex-, and calendar year-matched referents across seven age groups. Interestingly, it appears that younger patients have longer average lifetime employment duration. However, these younger patients also seem to experience a loss in lifetime employment duration when compared to their corresponding referents. The relative loss of lifetime employment duration, expressed in percentages, is as follows for the different age groups: 52% (54%), 55% (44%), 56% (42%), 53% (38%), 48% (36%), 43% (34%), and 32% (25%). The first figure in each pair refers to male patients, while the second figure refers to female patients. Figure 1 visually represents these findings. It shows the differences in the expected lifetime employment duration between male and female ESKD patients and their corresponding male and female referents. These differences are indicated by the light-gray area in the figure.

**Table 2:** The life expectancy (LE), the lifetime employment duration (LED), and the relative loss of LED for patients with end stage kidney disease (Index) and their corresponding age-, sex-, and calendar year-matched referents (Reference)

Group	Life expectancy (year)			LED (year)			Relative loss
	Index	Reference	Loss of LE	Index	Reference	Loss of LED	Loss of LED/Reference
**Male**							
Aged 25–34	28.7 (21.9–33.4)	47.9 (47.7–48.0)	19.2 (14.1–25.7)	11.6 (9.8–12.2)	24.0 (23.8–24.0)	12.4 (11.6–14.0)	52% (48–59%)
35–40	19.7 (15.2–21.3)	40.9 (40.7–41.0)	21.2 (19.2–25.4)	8.1 (7.3–8.4)	18.1 (18.1–18.2)	10.0 (9.6–10.8)	55% (53–60%)
41–45	14.7 (12.7–15.0)	35.8 (35.8–36.0)	21.1 (20.8–23.1)	6.2 (5.8–6.3)	14.0 (14.0–14.0)	7.8 (7.7–8.2)	56% (55–59%)
46–50	11.3 (10.4–11.3)	31.7 (31.3–31.7)	20.4 (20.3–21.0)	4.9 (4.7–4.9)	10.4 (10.3–10.4)	5.5 (5.4–5.7)	53% (52–55%)
51–55	9.6 (8.9–9.7)	27.5 (27.5–27.6)	17.9 (17.8–18.6)	3.6 (3.4–3.6)	6.9 (6.8–6.9)	3.3 (3.2–3.4)	48% (47–50%)
56–60	7.9 (7.5–7.9)	23.5 (23.2–23.5)	15.6 (15.5–15.9)	2.0 (1.9–2.1)	3.5 (3.5–3.6)	1.5 (1.5–1.6)	43% (41–45%)
61–64	7.0 (6.7–7.1)	19.9 (19.8–20.0)	12.9 (12.8–13.2)	0.7 (0.7–0.7)	1.0 (1.0–1.1)	0.3 (0.3–0.4)	32% (29–34%)
**Female**							
Aged 25–34	31.5 (21.4–36.5)	54.0 (53.8–54.1)	22.5 (17.1–31.5)	10.7 (9.2–11.1)	23.5 (23.4–23.6)	12.8 (12.4–14.2)	54% (52–61%)
35–40	25.1 (19.2–28.1)	46.6 (46.4–46.6)	21.5 (16.4–27.0)	9.8 (8.9–10.2)	17.6 (17.5–17.6)	7.8 (7.4–8.6)	44% (42–49%)
41–45	19.1 (16.6–20.4)	41.4 (41.3–41.5)	22.3 (18.9–24.8)	7.8 (7.4–8.0)	13.5 (13.5–13.6)	5.7 (5.5–6.2)	42% (41–46%)
46–50	16.0 (14.5–16.5)	36.7 (36.6–36.8)	20.7 (20.1–22.2)	6.1 (5.7–6.1)	9.8 (9.7–9.8)	3.7 (3.6–4.0)	38% (37–41%)
51–55	12.1 (11.1–12.5)	32.1 (32.0–32.2)	20.0 (19.6–20.9)	4.0 (3.8–4.1)	6.2 (6.1–6.2)	2.2 (2.1–2.4)	36% (34–39%)
56–60	9.6 (9.1–9.9)	27.4 (27.4–27.5)	17.8 (17.5–18.3)	2.0 (1.9–2.0)	3.0 (2.9–3.0)	1.0 (0.9–1.1)	34% (32–36%)
61–64	7.6 (7.4–7.8)	23.3 (23.2–23.4)	15.7 (15.6–15.9)	0.6 (0.6–0.6)	0.8 (0.8–0.8)	0.2 (0.2–0.2)	25% (22–28%)

### Estimation of lifetime productivity

**Fig. 2:**
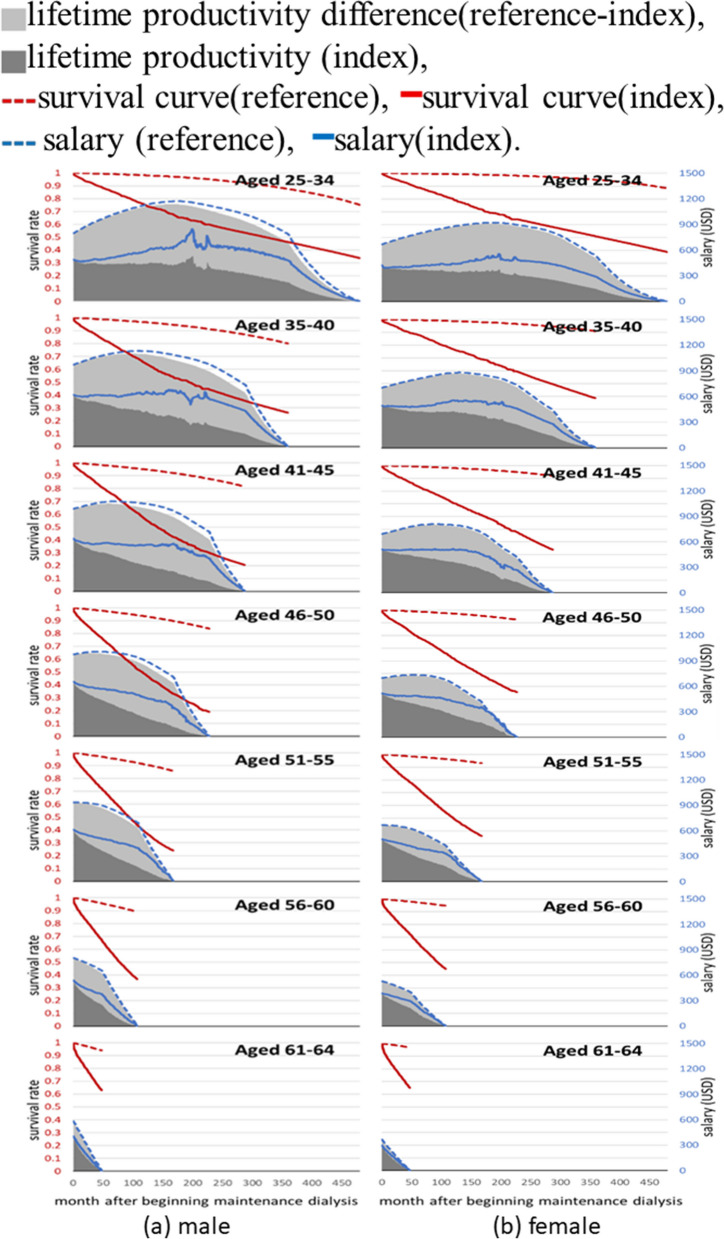
Expected lifetime productivity of male ESKD patients and corresponding age-sex-, and calendar year-matched referents, stratified by age

By multiplying the lifetime survival function and the monthly salary of the index cohort, we can calculate the lifetime earnings or productivity in USD for each of the seven age groups. Table 3 presents the results of this calculation and reveals the relative loss of lifetime productivity, expressed as a percentage for each age group. The percentages were as follows: 61% (61%), 63% (53%), 66% (52%), 64% (49%), 60% (47%), 55% (42%), and 44% (32%) (Fig. 2).

**Table 3:** The lifetime productivity (estimated by earnings) for patients with end stage kidney disease (ESKD) and their sex-, age-, and calendar year-matched referents, along with the relative loss of the index group

Group	Lifetime Productivity (in USD)		Foregone earnings (in USD)	Relative loss
	Index	Reference	Reference—Index	Foregone earnings/Reference
**Male**				
Aged 25–34	151,637 (123,093–163,971)	387,970 (386,059–388,912)	236,334 (222,645–263,113)	61% (57–68%)
Aged 35–40	109,220 (93,881–114,167)	297,409 (296,215–298,148)	188,189 (182,892–202,950)	63% (61–69%)
Aged 41–45	77,449 (70,523–79,523)	225,214 (224,683–225,945)	147,765 (145,748–154,131)	66% (65–69%)
Aged 46–50	58,314 (55,265–59,676)	163,907 (163,176–164,086)	105,593 (103,698–108,174)	64% (63–66%)
Aged 51–55	42,222 (40,145–43,191)	106,023 (105,570–106,268)	63,801 (62,555–65,445)	60% (59–62%)
Aged 56–60	23,188 (22,310–23,882)	51,958 (51,721–52,201)	28,771 (27,954–29,622)	55% (54–57%)
Aged 61–64	7,777 (7,468–8,110)	13,949 (13,784–14,144)	6,173 (5,829–6,425)	44% (41–47%)
**Female**				
Aged 25–34	120,035 (98,995–127,596)	311,230 (309,814–312,478)	191,195 (181,497–211,648)	61% (58–68%)
Aged 35–40	107,202 (96,897–110,424)	230,081 (228,803–230,888)	122,879 (119,230–132538)	53% (52–58%)
Aged 41–45	81,621 (76,597–84,070)	171,502 (170,676–172,250)	89,881 (87,120–94,302)	52% (51–55%)
Aged 46–50	61,464 (57,861–62,758)	119,905 (119,329–120,319)	58,442 (56,722–61,452)	49% (47–51%)
Aged 51–55	39,243 (37,521–40,410)	73,917 (73,491–74,242)	34,673 (33,449–36,263)	47% (45–49%)
Aged 56–60	19,361 (18,786–19,971)	33,474 (33,256–33,711)	14,113 (13,334–14,641)	42% (40–44%)
Aged 61–64	5,685 (5,457–5,901)	8,391 (8,293–8,519)	2,706 (2,441–2,865)	32% (29–35%)

**Fig. 3:**
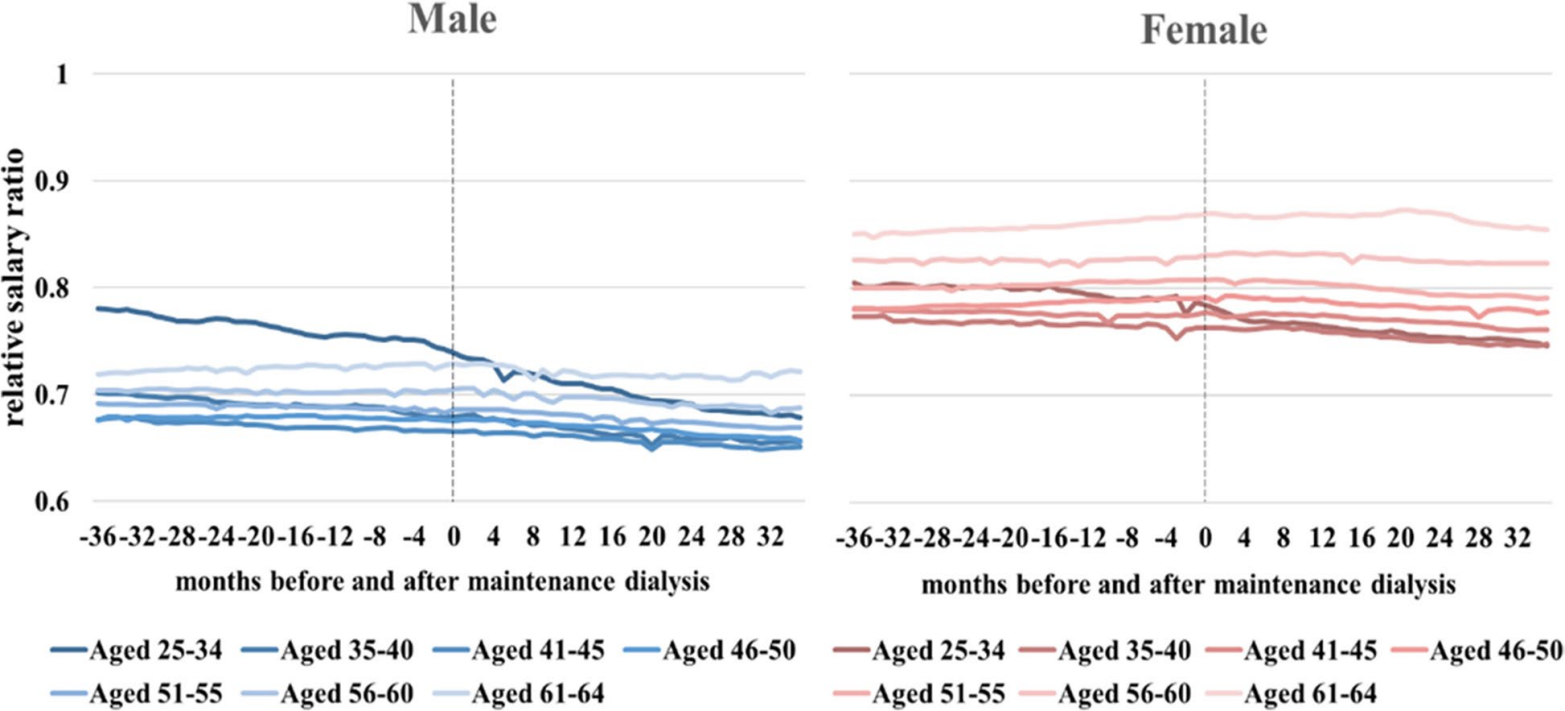
The ratio of average salaries between patients with ESKD (end stage kidney disease) and corresponding age-, sex-, and calendar year-matched referents for 3 years before and after dialysis stratified by sex and age

Figure 3 provides a visual representation of these findings, illustrating the differences in expected lifetime productivity between male and female ESKD patients and their corresponding male and female referents. The differences are indicated by the light-gray area in the figure.

The decrease in lifetime productivity is a combined result of several obvious factors including: reduced lifetime employment duration, absenteeism, and presenteeism due to the underlying causes of increased premature mortality and functional disability. We used the data from Tables 2 and 3 to calculate the annual earnings (which is lifetime earnings divided by lifetime employment duration in years) as shown in Supplementary Table 1. We then directly compared these annual earnings for the ESKD cohort with those of the corresponding reference population. The ratio of the annual earnings of the index cohort to that of the corresponding referents is equivalent to the ratio of the patients’ relative lifetime productivity to their relative lifetime employment duration. The productivity of each male (female) ESKD patient, relative to that of the age-, sex-, and calendar year-matched referent, ranges from 75.5% to 82.1% (82.3% to 90.3%) across the seven age strata. However, during the on-the-job duration, these percentages are between 34 and 56% for male patients and between 39 and 68% for female patients. A consistent reduction in annual earnings was observed in each stratum as shown in Supplementary Table 1. This seems to capture the productivity loss due to presenteeism resulting from ESKD.

## Discussions

This research investigates the hypothesis that individuals suffering from chronic catastrophic illnesses experience a lifetime loss of working productivity. Previous studies focused solely on temporary absence from work or mortality resulting from illness [5, 7]. We considered a combination of factors including premature death, temporary absence from work, and presenteeism at work. Using real-world data, we estimated the losses in both lifetime employment duration and earnings. While we identified negative correlation between illness and earnings, this does not necessarily indicate that productivity loss is solely caused by illness or presenteeism. Various pieces of evidence support our hypothesis. Firstly, we compared the average salaries of male and female ESKD patients with those of the general population (matched for age, sex, and calendar year) for three consecutive years before and after dialysis. Employed patients with chronic kidney failure had lower average wages than their corresponding referents even before dialysis. This suggests a chronic loss of productivity, with this effect being more pronounced in males and younger age groups (males under 50 and females under 40) [17]. Secondly, there was a downward trend in earnings compared to the corresponding referents after patients began dialysis at the end stage. We can even conclude that presenteeism may predict a lifetime productivity loss at the workplace due to a bad state of health. However, this trend appears to have been less severe among middle-aged and older patients.

Researchers have been focusing on presenteeism and its association with various chronic illnesses [18]. However, there is currently no universally accepted method for measuring presenteeism [19, 20]. FCM and HCM are used to estimate productivity loss due to short- and long-term employee absenteeism, respectively. However, there is a lack of evidence on their validity in evaluating lifetime productivity loss due to illness. This study addresses this gap by the lifetime survival function of ESKD patients up to the age of 65, their employment status, and income.

Patients with Chronic Kidney Disease (CKD) or End Stage Kidney Disease (ESKD) commonly experience protein-energy wasting, cardiovascular and cerebrovascular diseases [21–23]. These factors contribute in a cumulative manner to physical and mental disabilities that impair productivity at the workplace. The implementation of CKD preventive strategies in clinical practice has potential economic benefits as evidenced by studies. These studies estimate that a 10% reduction in CKD patients could result in a saving of USD 1.1 billion in gross domestic product over a ten-year period in Australia [24]. Similarly, a 10% decrease in the glomerular filtration rate decline in CKD patients could lead to a saving of USD 18.56 billion in direct healthcare expenditure over 10 years in the United States [25]. Therefore, these studies and ours all highlight the substantial benefit to integrating the CKD preventive strategies into clinical practice, which would save both the potential productivity loss and healthcare expenditure related to CKD.

The previous studies assumed that human capital depreciated at a constant rate over time [26–28]. Our study proposed a novel depreciation mechanism for the human capital over time. Different illnesses bring about varying degrees of presenteeism and labor-market participation rates, hence prevention strategies could be varied. More accurate estimations of such effects would enhance the incentive for prevention investment and proper allocations of healthcare resources [29, 30].

### Limitation of this study

This study has following limitations: Firstly, the insurance premium collected by the Taiwan NHI is based on the monthly salary, excluding bonuses and other forms of compensation. This could lead to an underestimation of the real income for both the index cohort and referents [31]. Bonuses, often linked to productivity can exceed monthly salaries [32, 33]. Lower salaries are typically associated with lower bonuses [34]. Even though, the healthy referents have higher wages and bonus, but are also required to pay higher tax than ESKD patients. Therefore, we estimated the relative loss of lifetime productivity. Secondly, in non-competitive labor markets, productivity is likely to exceed wages. Even in a perfectly competitive market, wages would only equate to marginal productivity, not average productivity [35]. As there were 83,358 eligible ESKD patients with different wages included in this study and many of them were out-of-job, or no salary, this study tried to quantify the lifetime productivity loss of a representative individual. Therefore, we chose to use the average productivity instead of marginal productivity in order to avoid making extreme inferences based on higher or lower marginal productivity [36]. Fortunately, from the societal perspective, the summation of all patients’ marginal productivities could be equivalent to that of average productivities [37]. Thirdly, though non-market output plays a significant role in estimating productivity loss, this study did not consider this factor due to limited data availability. This research did underestimate the productivity loss but filled in the void of previous studies through estimating the lifetime productivity loss of patients with chronic diseases from a societal perspective.

## Conclusions and policy implications

This research delved into the impact of end stage kidney disease on an individual’s productivity. Specifically, we discovered a loss of about 25–56% in lifetime employment duration and a more significant loss of about 32–66% in lifetime productivity after accounting for variations in age, sex, and calendar years. The authors suggest that their approach, which integrates survival, employment ratios, and earnings, provides a more comprehensive way to evaluate the societal value of health interventions. They encourage further research to apply this approach to other serious illnesses.

Traditionally, health economists have focused on the costs of healthcare, particularly as these costs have been rising rapidly since the 1960s. However, this study argues that it is also important to consider the impact of health on productivity, particularly for people who live with serious illnesses like end stage kidney disease. The authors propose a “positive feedback loop” between individual engagement in the labor market and societal benefits [38]. In other words, when individuals with health issues are able to continue working, this is not only benefits them personally through income, a sense of purpose, etc., but also benefits society as a whole by contributing to overall productivity and economic output.

### Supplementary Information

**Additional file 1: S. Table 1. **The estimated annual earnings (in USD) of the index and reference groups and the relative ratios between them stratified by sex and age.

**Additional file 2: S. Figure 1. **The ratio of the employed to the civilian population (EMRATIO) stratified by sex, age and calendar year (2000-2017).

**Additional file 3: S. Figure 2.** The average salary (the total salary/the civilian population) of the employed stratified by sex, age and calendar year (in USD) (2000-2017).

## Data Availability

Data are available from the Institutional Review Board(IRB)of the National Cheng Kung University Hospital (NCKUH) for researchers who meet the criteria for access to confidential data for academic purposes with a permission letter. The IRB of NCKUH are entitled and have full rights to oversee all activities, including the data of this study of each research to comply with the Personal Data Protection Act. Anyone who is interested in analyzing the same dataset must write a research proposal with full protection of human rights and apply to the IRB of NCKUH to get access. Researchers may obtain a permission letter for requesting data from the following website: http://nckuhirb.med.ncku.edu.tw/. They usually would not turn down an application with approval from a credible Institutional Review Board or ethics committee.

## Abbreviations

ESKD: End-stage kidney disease
HCM: Human capital method
FCM: Friction cost method
EMRATIO: The ratio of the employed to the civilian population
NHI: National Health Insurance
CKD: Chronic kidney disease

## References

[1] Sanders GD, Neumann PJ, Basu A, Brock DW, Feeny D, Krahn M, Kuntz KM, Meltzer DO, Owens D, Prosser LA, Saloman JA, Sculpher MJ, Trikalinos TA, Russell LB, Siegel JE, Ganiats TG (2016). Recommendations for conduct, methodological practices, and reporting of cost-effectiveness analyses: second panel on cost-effectiveness in health and medicine. JAMA.

[2] Neumann PJ, Ganiats TG, Russell LB, Sanders GD, Siegel, JE. Cost-effectiveness in health and medicine. New York: Oxford University Press; 2016.

[3] Neumann PJ, Garrison LP, Willke RJ (2022). The history and future of the “ISPOR value flower”: addressing limitations of conventional cost-effectiveness analysis. Value Health.

[4] Chang YT, Wang F, Huang WY, Hsiao H, Wang JD, Lin CC (2021). Estimated loss of lifetime employment duration for patients undergoing maintenance dialysis in Taiwan. Clin J Am Soc Nephrol.

[5] Pike J, Grosse SD (2018). Friction cost estimates of productivity costs in cost-of-illness studies in comparison with human capital estimates: a review. Appl Health Econ Health Policy.

[6] Lo JC (2019). Employment pathways of cancer survivors-analysis from administrative data. Eur J Health Econ.

[7] van den Hout Wilbert B (2010). The value of productivity: human-capital versus friction-cost method. Ann Rheum Dis.

[8] Koopmanschap MA, Rutten FF, van Ineveld BM, van Roijen L (1995). The friction cost method for measuring indirect costs of disease. J Health Econ.

[9] Krol M, Brouwer W (2014). How to estimate productivity costs in economic evaluations. Pharmacoeconomics.

[10] Rissanen I, Ala-Mursula L, Nerg I, Korhonen M (2021). Adjusted productivity costs of stroke by human capital and friction cost methods: a Northern Finland Birth Cohort 1966 study. Eur J Health Econ.

[11] Hwang JS, Tsauo JY, Wang JD (1996). Estimation of expected quality adjusted survival by cross-sectional survey. Stat Med.

[12] Hwang JS, Hu TH, Lee LJ, Wang JD (2017). Estimating lifetime medical costs from censored claims data. Health Econ.

[13] van Oostrum I, Ouwens M, Remiro-Azócar A, Baio G, Postma MJ, Buskens E, Heeg B (2021). Comparison of parametric survival extrapolation approaches incorporating general population mortality for adequate health technology assessment of new oncology drugs. Value Health.

[14] Becker GS (1962). Investment in human capital: A theoretical analysis. J Polit Econ.

[15] Chung CH, Hu TH, Wang JD, Hwang JS (2020). Estimation of quality-adjusted life expectancy of patients with oral cancer: Integration of lifetime survival with repeated quality-of-life measurements. Value Health Reg Issues.

[16] Department of Statistics. Vital statistics. The Ministry of Interior, Executive Yuan, Taiwan; 2022. https://www.moi.gov.tw/stat/life.aspx. Accessed 16 June 2022.

[17] Crews Deidra C, Gutiérrez Orlando M, Fedewa Stacey A, Luthi JC, Shoham D, Judd Suzanne E, Powe Neil R, McClellan William M (2014). Low income, community poverty and risk of end stage renal disease. BMC Nephrol.

[18] Schultz AB, Edington DW (2007). Employee health and presenteeism: a systematic review. J Occup Rehabil.

[19] Bierlaa I, Huverb B, Richardb S (2013). New evidence on absenteeism and presenteeism. Int J Human Resour Manag.

[20] Ishimaru T, Mine Y, Fujino Y (2020). Two definitions of presenteeism: sickness presenteeism and impaired work function. Occup Med.

[21] Chang YT, Wu JL, Hsu CC, Wang JD, Sung JM (2013). Diabetes and end-stage renal disease synergistically contribute to increased incidence of cardiovascular events: a nationwide follow-up study during 1998–2009. Diabetes Care.

[22] Ikizler A, Cano TNJ, Franch H, Fouque D, Himmelfarb J, Kalantar-Zadeh K, Kuhlmann MK, Stenvinkel P, TerWee P, Teta D, A. Wang YM, Wanner C (2013). Prevention and treatment of protein energy wasting in chronic kidney disease patients: a consensus statement by the International Society of Renal Nutrition and Metabolism. Kidney Int.

[23] Jankowski J, Floege J, Fliser D, Böhm M, Marx N (2021). Cardiovascular disease in chronic kidney disease. Circulation.

[24] Savira F, Ademi Z, Wang BH, Kompa AR, Owen AJ, Liew D, Zomer E (2021). The preventable productivity burden of kidney disease in Australia. J Am Soc Nephrol.

[25] Trivedi H (2010). Cost implications of caring for chronic kidney disease: are interventions cost-effective?. Adv Chronic Kidney Dis.

[26] Hashmi AR (2013). Intangible capital and international income differences. Macroecon Dyn.

[27] Ortigueira S (1998). Fiscal policy in an endogenous growth model with human capital accumulation. J Monet Econ.

[28] Roufagalas J, Orlov AG (2020). Endogenous growth, human capital and the dynamic costs of recessions. J Econ Stud.

[29] Wang F, Wang JD, Huang YX (2016). Health expenditures spent for prevention, economic performance, and social welfare. Heal Econ Rev.

[30] Wang F, Wang JD (2021). Investing preventive care and economic development in ageing societies: empirical evidences from OECD countries. Heal Econ Rev.

[31] Lien HM (2011). How to construct social-economic variables from National Health Insurance data. J Soc Sci Philos.

[32] Krueger AB, Summers LH (1988). Efficiency wages and the interindustry wage structure. Econometrica.

[33] Biesebroeck JV (2011). Wages equal productivity: fact or fiction? Evidence for Sub-Saharan Africa. World Dev.

[34] Borjas GJ (2010). Labor Economics.

[35] Kaufman BE (2007). The impossibility of a perfectly competitive labour market. Camb J Econ.

[36] Biesebroeck JV. Wage and productivity premiums in Sub-Saharan Africa. In Bender S, Lane J, Shaw KL, Andersson F, von Wachter T, editors. The analysis of firms and employees: quantitative and qualitative approaches. Chicago: University of Chicago Press; 2008.

[37] Hamermesh DS, Ashenfelter O, Layard R (1986). The demand for labor in the long run. Handbook of labor economics.

[38] Medicine NAO (2022). Global roadmap for healthy longevity.

