# Supplementary material for: Estimation of lifetime productivity loss from patients with chronic diseases: methods and empirical evidence of end-stage kidney disease from Taiwan
Source: Health Econ Rev. 2024 Feb 6;14:10. doi: 10.1186/s13561-024-00480-z (PMC10848535; doi:10.1186/s13561-024-00480-z)
Supplement: Supplementary file 1 — Additional file 1: S. Table 1. The estimated annual earnings (in USD) of the index and reference groups and the relative ratios between them stratified by sex and age. [file 13561_2024_480_MOESM1_ESM.docx]

**S. Table 1.** The estimated annual earnings (in USD) of the index and reference groups and the relative ratios between them stratified by sex and age

| Group | **Index** | **Referents** | **Ratio (Index/Referents: %)** |
| --- | --- | --- | --- |
| **Male** |  |  |  |
| Aged 25-34 | 13072 | 16165 | 80.9 |
| Aged 35-40 | 13484 | 16431 | 82.1 |
| Aged 41-45 | 12492 | 16087 | 77.7 |
| Aged 46-50 | 11901 | 15760 | 75.5 |
| Aged 51-55 | 11728 | 15366 | 76.3 |
| Aged 56-60 | 11594 | 14845 | 78.1 |
| Aged 61-64 | 11110 | 13949 | 79.7 |
| **Female** |  |  |  |
| Aged 25-34 | 11218 | 13244 | 84.7 |
| Aged 35-40 | 109384 | 13073 | 83.7 |
| Aged 41-45 | 10464 | 12704 | 82.4 |
| Aged 46-50 | 10076 | 12235 | 82.4 |
| Aged 51-55 | 9811 | 11922 | 82.3 |
| Aged 56-60 | 9681 | 11158 | 86.8 |
| Aged 61-64 | 9475 | 10488 | 90.3 |
