# Supplementary figures and images for: Estimation of lifetime productivity loss from patients with chronic diseases: methods and empirical evidence of end-stage kidney disease from Taiwan
Source: Health Econ Rev. 2024 Feb 6;14:10. doi: 10.1186/s13561-024-00480-z (PMC10848535; doi:10.1186/s13561-024-00480-z)

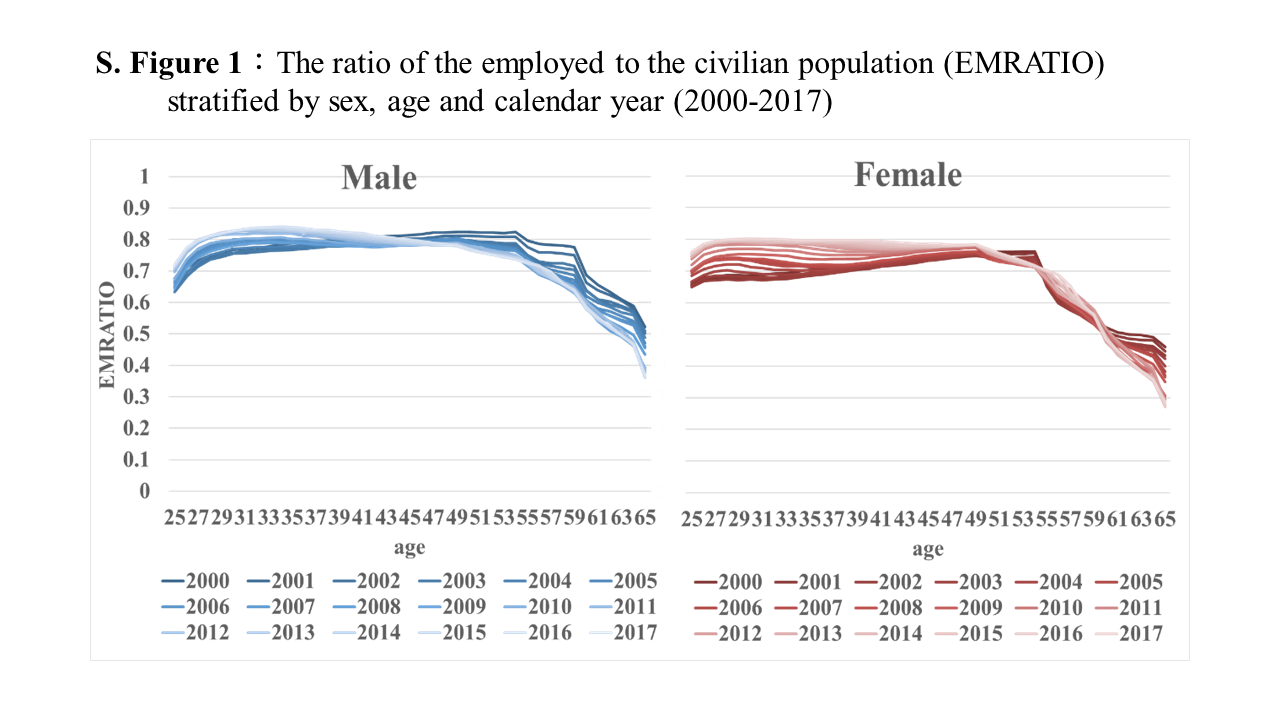

Supplement: Supplementary file 2 — Additional file 2: S. Figure 1. The ratio of the employed to the civilian population (EMRATIO) stratified by sex, age and calendar year (2000-2017). [file 13561_2024_480_MOESM2_ESM.tif]

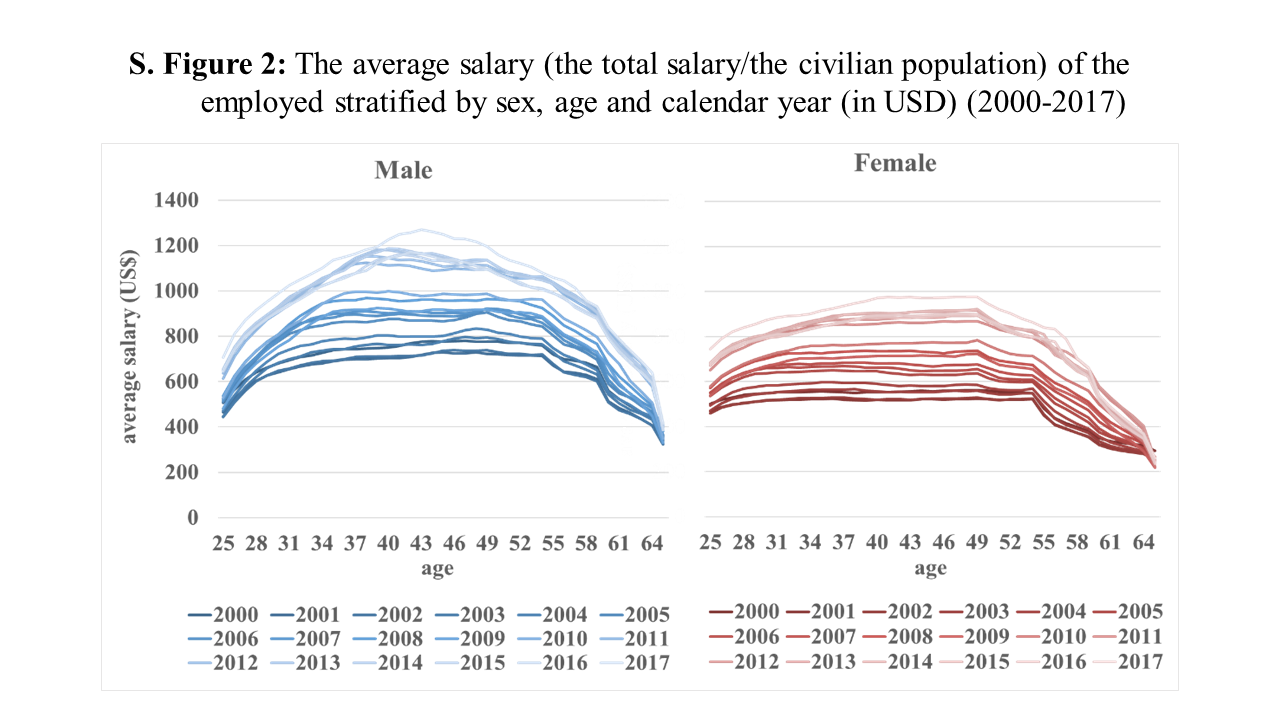

Supplement: Supplementary file 3 — Additional file 3: S. Figure 2. The average salary (the total salary/the civilian population) of the employed stratified by sex, age and calendar year (in USD) (2000-2017). [file 13561_2024_480_MOESM3_ESM.tif]
